# Defining exceptional cognition in older adults: A systematic review of cognitive super‐ageing

**DOI:** 10.1002/gps.6034

**Published:** 2023-12-11

**Authors:** Alice Powell, Zara A. Page, Jacqueline C. T. Close, Perminder S. Sachdev, Henry Brodaty

**Affiliations:** ^1^ Centre for Healthy Brain Ageing Discipline of Psychiatry and Mental Health School of Clinical Medicine University of New South Wales Randwick New South Wales Australia; ^2^ Neuroscience Research Australia University of New South Wales Sydney New South Wales Australia; ^3^ The Prince of Wales Hospital Clinical School University of New South Wales Sydney New South Wales Australia; ^4^ Neuropsychiatric Institute The Prince of Wales Hospital Clinical School University of New South Wales Sydney New South Wales Australia

**Keywords:** cognitively elite, exceptional cognition, high performing older adult, successful ageing, super normal, super‐ageing

## Abstract

**Objective:**

A consistent approach to defining cognitive super‐ageing is needed to increase the value of research insights that may be gained from studying this population including ageing well and preventing and treating neurodegenerative conditions. This review aims to evaluate the existing definitions of ‘super‐ageing’ with a focus on cognition.

**Methods:**

A systematic literature search was conducted across PubMed, Embase, Web of Science, Scopus, PsycINFO and Google Scholar from inception to 24 July 2023.

**Results:**

Of 44 English language studies that defined super‐ageing from a cognitive perspective in older adults (60–97 years), most (*n* = 33) were based on preserved verbal episodic memory performance comparable to that of younger adult in age range 16–65 years. Eleven studies defined super‐agers as the top cognitive performers for their age group based upon standard deviations or percentiles above the population mean. Only nine studies included longitudinal cognitive performance in their definitions.

**Conclusions:**

Equivalent cognitive abilities to younger adults, exceptional cognition for age and a lack of cognitive deterioration over time are all meaningful constructs and may provide different insights into cognitive ageing. Using these criteria in combination or individually to define super‐agers, with a clear rationale for which elements have been selected, could be fit for purpose depending on the research question. However, major discrepancies including the age range of super‐agers and comparator groups and the choice of cognitive domains assessed should be addressed to reach some consensus in the field.

## Key points

- There is no uniform definition of super‐ageing or cognitive super‐ageing
- Most studies define cognitive super‐ageing based on verbal memory
- Proposed definition should include other cognitive domains, stability over time and function
- An agreed definition would support a collaborative approach for associated factors and studying this group may highlight strategies to combat cognitive decline

## 1 INTRODUCTION

A major goal of ageing research is to identify factors associated with a delay in the emergence of age‐related disease and a lower burden of disease to promote healthy life expectancy or ‘health‐span’.^1^ Delaying the negative impacts of ageing and promoting health in later life is related to the construct of successful ageing which broadly comprises interrelated components of high cognitive and physical capacity and active social engagement.^2^ These domains are interrelated with higher social participation and higher levels of physical activity associated with better cognitive function.^3^, ^4^ Successful cognitive ageing, more recently termed ‘super‐ageing’^5^ and how this is measured will be the focus of this review.

Ageing has been examined through changes in bodily processes and organ systems and has been measured by changes observed clinically in cognitive processes, body composition, physical and sensory function or the quantification of frailty.^6^, ^7^ Biomarkers can also be used to measure ageing such as DNA methylation or so‐called ‘epigenetic clocks’, replicating proteins associated with chronological age or proteomics, telomere length, metabolites/metabolomics and neuroimaging markers.^6^, ^7^ These markers may also allow for the identification of discrepancies between biological and chronological age.

Super‐ageing has been used to describe individuals who maintain midlife levels of function and activity into advanced ages. A group of older adults who maintain physical activity levels and the aerobic capacity of much younger individuals could be considered physical super‐agers.^8^, ^9^ More recently, this term has been applied to cognitive abilities.^5^, ^10^ Cognitive super‐agers (henceforth ‘super‐agers’) have been shown to have higher occupational attainment,^11^ a musical background,^12^ lower rates of smoking and diabetes and exhibit higher levels of intellectual and social activity than their cognitively average peers.^11^, ^13^, ^14^ The association between years of education and super‐ageing has not been consistent.^11^, ^12^, ^15^ Super‐agers have been shown to be resilient to post‐operative delirium.^16^ Genetic differences include lower rates of the apolipoprotein ε4 allele which is associated with an increased risk of Alzheimer's disease (AD)^17^ as well as variants in the Mitogen‐Activated Protein Kinase 3 (MAP2K3) gene.^18^ Imaging studies of the brains of super‐agers have demonstrated less cortical atrophy, differences in functional connectivity, greater white matter integrity and a reduced burden of positron emission tomography (PET) amyloid and tau compared with control participants.^5^, ^19^, ^20^, ^21^, ^22^ Neuropathological studies have reported less but still significant Alzheimer's type pathology.^17^, ^23^, ^24^ This suggests that some factors associated with super‐ageing status are modifiable and that mechanisms of both resistance and resilience to neurodegenerative pathology involving brain reserve and cognitive reserve processes^25^ may underly exceptional cognition at older ages. Identifying super‐agers and modifiable contributors to super‐ageing has the potential to inform strategies to prevent development of dementia and maintain cognitive health across the lifespan. This may have wide‐ranging benefits for individuals and broader society.

How super‐ageing is best defined and how it differs from usual or normative ageing remains unanswered. Several approaches have been taken to categorise super‐agers, for example, comparing the performance of older adults on tests assessing one or more cognitive domains with middle aged or younger adults or selecting the top cognitive performers from a sample of age peers.^5^, ^19^ Episodic memory performance has frequently been selected as a marker of cognitive super‐ageing^5^, ^26^ with executive function, processing speed and composite cognitive scores less commonly utilised.^14^, ^27^, ^28^ Some studies have also identified individuals without cognitive decline over time. These approaches have thus far been relatively limited regarding how superior cognitive performance relates to multidimensional biological ageing parameters, physical capacity or functioning in the community.

There have been two reviews of super‐ageing^26^, ^29^ and one review examining the cognitive component of successful ageing.^30^ A narrative review focussed upon operationalised definitions^26^ and a systematic review on biomarkers.^29^ No studies have yet compared definitions or rating methods. This review aims to systematically evaluate the literature identifying older adults with exceptional cognitive performance with emphasis on how super‐ageing is defined and the key clinical features that distinguish this group from the general older adult population. The main focus is upon definitions of exceptional cognitive performance including cut‐off scores on neuropsychological tests and key cognitive domains. How superior cognitive performance relates to other aspects of exceptional ageing including physical, functional and social capacity will also be considered.

## 2 MATERIALS AND METHODS

The study protocol was registered with PROSPERO (CRD42021234387) prior to commencement of the study. Processes for article inclusion and data analysis followed PRISMA guidelines.^31^

### 2.1 Search strategy

A systematic review of the literature was performed through searches of the following databases: PubMed (including MEDLINE), Embase, Web of Science, Scopus, PsycINFO and Google Scholar from inception to 9th December 2021. Searches were updated in 2022 and 2023. The search strategy included a comprehensive list of terms related to the broad concept of successful or super ageing (older people OR older person × OR older adult × OR aged person × OR aged people OR elderly OR senior citizen OR senior OR geriatric OR aged AND super‐ageing OR super‐ageing OR superaging OR superageing OR supernormal × OR successful ageing OR successful ageing OR ageing successfully OR ageing successfully OR ageing well OR ageing well OR successful agre × OR super‐ager × OR exceptional ageing OR exceptional ageing OR high performing older adult × OR healthy ageing) and exceptional cognition (exceptional cognition OR exceptional cognitive OR successful cognitive OR higher cognitive OR high performing OR memory OR episodic memory OR exceptional memory OR exceptional memory capacity OR excellent cognition OR excellent memory OR frontal function OR cognition OR memory OR working memory OR attention OR executive function OR activity, higher nervous OR perception OR spatial navigation OR thinking OR theory of mind OR language). The search term clusters were developed through review of prior systematic and narrative reviews focussed on super‐ageing. The search strategy was designed to capture all studies concerning exceptional ageing in regards to cognition.

### 2.2 Study screening and selection

The abstracts of all articles were screened for eligibility criteria by two authors (AP and ZP) with disagreement resolved through review of the full‐text or discussion with a third author. These criteria included English language studies concerning older adults (age 65 and above) deemed to be ageing well compared to the general population with reference to cognitive health. Articles were excluded at the screening stage if they contained no reference to super‐ageing, successful ageing or related terms or were duplicates, full text was not available in English, animal studies, books or book reviews, theses/dissertations or only abstracts from conference proceedings with no full text manuscript available. The full texts of the remaining articles were obtained. Relevant articles in the reference lists of included studies were also sourced and added to the sample. Full‐text articles were examined for definitions of super‐ageing and related concepts, type of study and population characteristics by the first author (AP).

### 2.3 Risk of bias assessment and methodologic quality assessment

Two review authors (AP and ZP) independently assessed risk of bias in the core analysis group of studies using checklists from the Joanna Briggs Institute (JBI) Manual for Evidence Synthesis^32^ relevant to the individual study design. These results were reported; JBI quality ratings were not used to exclude any study which provided an explicit definition of super‐ageing.

### 2.4 Data synthesis, extraction and analysis

For each included study in the core analysis group, the following data were extracted: author details, study type, study population and how this was derived, age of participants, definition of super‐ageing used, comparator group/s, cognitive tests and rating scales used and any biomarkers. Measures of cognition used to define super‐agers were split into global cognitive screening tests such as the Mini‐Mental State Examination (MMSE) and clinician or informant rating scales such as the Clinical Dementia Rating Scale (CDR), as well as measures of specific cognitive domains incorporated in neuropsychological batteries. The variability of cognitive tests used and paucity of quantitative data precluded meta‐analysis.

## 3 RESULTS

**FIGURE 1:**
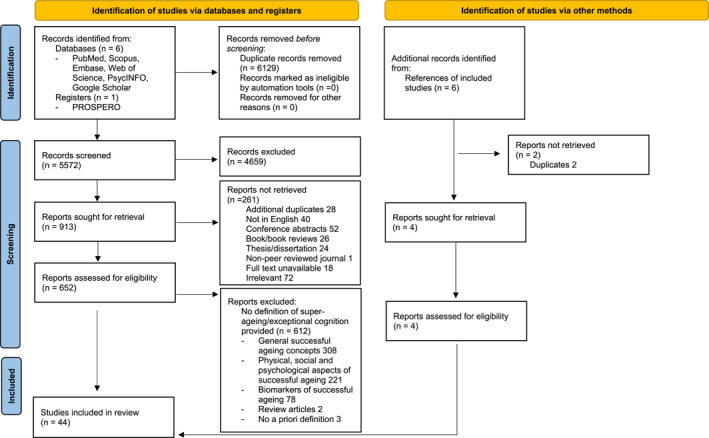
PRISMA flow diagram of study selection and reasons for exclusion.

Of 656 articles identified (652 from the search process and an additional 4 from searching reference lists of retrieved articles), 44 were included in the final analysis. Figure 1 depicts the PRISMA‐based flow chart of study selection and reasons for exclusion.^33^ The study designs, population, details on super‐ageing definition, comparator population/s, cognitive tests used and any biomarkers measured are presented in Tables 1 and 2. There were 27 cross‐sectional studies (case‐control), 15 cohort studies and 2 case series. The reference lists of included studies and reviews (*n* = 2) were scanned for additional studies that met inclusion criteria.

**TABLE 1:** Super‐ageing studies.

Author, year	Study population, derivation and design	Participants and comparators (number, mean age ± SD)	Inclusion criteria	Biomarkers
Baran and Lin 2018^34^	ADNI multisite (US and Canada) longitudinal study recruiting 55–90yo Longitudinal, cognition assessed over 5 years, single imaging session	‘Supernormals’ (*n* = 122, 73.88 ± 6.64) AD (*n* = 27, 73.18 ± 7.34), MCI (*n* = 69, 71.27 ± 7.84), NC (*n* = 172, 74.56 ± 6.17)	NC and ‘supernormals’ free of MCI, any type of dementia or major psychiatric disorder, MMSE ≥24, CDR 0	T1 weighted MRI, amyloid PET, FDG‐PET
Bezdicek et al 2020^35^	≥60yo volunteers in extension of Czech National normative study of cognitive Determinants of healthy ageing (NANOK) longitudinal study, annual cognitive assessments for 5 years and assessment of functional capacity	‘Successful healthy agers’ (*n* = 25, 74.9 ± 7.0) ‘Decliners’ (*n* = 15, 75.9 ± 4.8): Negative random slope on MoCA scores over 5 years	Independently living with no neurodegenerative disease, traumatic brain injury, stroke, substance abuse, epilepsy, major psychiatric disorder, unstable medical illness, current radiotherapy or chemotherapy or uncorrected visual or hearing impairment	Volumetric MRI
Biswas et al 2023^36^	≥90yo volunteers mostly from a retirement community in California, US, enroled in the 90+ (longitudinal) study with normal cognition who had at least one assessment within 2–12 months of death and had undergone autopsy	‘Superior global cognitive performers’ (*n* = 71, 97.4 ± 3.4) ‘Non‐superior global cognitive performers’ (*n* = 31, 98 ± 2.9)	No or mild dementia	Neuropathology
Borelli et al 2021^37^	50‐65yo and ≥80yo Brazilian community‐dwelling volunteers recruited through social media, television advertisements and university courses Cross‐sectional single assessment	‘SuperAgers’ (*n* = 10, 82.1 ± 2.5) Healthy controls in their 80s (*n* = 10, 84.2 ± 3.6) and healthy controls in their 50s (*n* = 10, 58.5 ± 5.8)	No history of substance abuse, significant head trauma, neurological or psychiatric disease, no family history of cognitive impairment, geriatric Depression scale scores <5 and preserved activities of daily living (function)	FDG‐PET, amyloid PET and structural and functional MRI
Bott et al 2017^27^	Convenience sample from existing research cohorts at the University of California San Francisco, US, memory and ageing Centre based on availability of cognitive processing speed data Longitudinal with two assessments, mean follow up 2.5 years. Association with genetic, inflammatory, cardiovascular, lifestyle and neuroanatomical factors.	‘Resilient‐agers’ (*n* = 17, 69.2 ± 0.96) Average‐agers (*n* = 56, 70.9, SE 0.65), sub‐agers (*n* = 47, 72.0, SE 0.68)	No neurologic or psychiatric disorders, sensory or motor disability, significant systemic illness, medications likely to affect CNS function or substance abuse. Independent daily function. CDR 0. MMSE >25	Genotyping—APOE and CR1 Blood interleukin‐6, lipid profile, fasting insulin Volumetric MRI
Cabeza et al 2002^38^	Not stated, Canadian sample Cross‐sectional single assessment, imaging	‘Old‐high’ (*n* = 8, 68 ± 4.1) Young participants (*n* = 12, 25.3 ± 4.1) and ‘old‐low’ (memory performance significantly below that of young adults; *n* = 8, 69.9 ± 3.7)	No history of neurological or psychiatric illness	PET
Carmona et al., 2023^15^	≥75yo living in Minas Gerais, Brazil identified from census records and invited to participate in the Pieta study. Aimed to identify high‐performing individuals with low education and associations with sociodemographic, clinical and lifestyle factors. Cross‐sectional single assessment.	‘High performance older adults’ (*n* = 18, 77.4 ± 2.6) ‘Standard performance older adults’ (*n* = 79.8 ± 4.5)	Cognitively healthy, no neurological or psychiatric conditions	Nil
Cervenkova et al 2020^39^	≥60yo participants in the Czech NANOK study Longitudinal cognitive trajectories, annual assessments for 3 years	‘SuperAgers’ (*n* = 20) Non‐SuperAgers (*n* = 81)	No cognitive impairment, neurologic or psychiatric illness, other serious medical illness or substance abuse	Nil
Cook et al 2017^40^	As per Harrison et al 2012 Two imaging sessions approximately 18 months apart for volume change	‘SuperAgers’ (*n* = 24, 83.3 ± 3.5) Cognitively average older adults (*n* = 12, 83.4 ± 3.8)	No known cognitive impairment, intact daily functioning, stable cognition between study visits	Structural MRI
Cook Maher et al 2017^41^	As per Harrison et al 2012 Cross‐sectional single assessment, assessment of psychological wellbeing	‘SuperAgers’ (*n* = 31, 83.4) Cognitively average older adults (*n* = 19, 84.4)	No neurological or psychiatric illness, preserved function	Nil
Cook Maher et al., 2022^42^	As per Harrison et al 2012 Retrospective analysis of neuropsychological test data to establish profiles of SA	‘SuperAgers’ (*n* = 56, 82.5 ± 2.6) Average‐memory older adults (*n* = 23, 83.5 ± 4.3)	English‐speaking, no neurological or psychiatric illness	Nil
Dang et al 2019^43^	Volunteer participants from the multisite Australian imaging, biomarkers and lifestyle (AIBL) longitudinal study over 8 years. Risk of progression to MCI or dementia	‘SuperAgers’ (*n* = 179, 68.48) Cognitively normal for age individuals (*n* = 179; 68.53)	No neurological or psychiatric illness, cancer in last 2 years, uncontrolled diabetes or other unstable medical condition, significant head injury, sleep apnoea, alcohol intake >4 SD per day for men and >2 for women	APOE genotype Amyloid‐beta PET
de Godoy et al 2021^44^	Volunteers recruited through clinics, newspaper and social media campaigns in Sao Paulo, Brazil Cross‐sectional single assessment investigating brain metabolite concentrations and late‐life cognition	‘Superagers’ (*n* = 12 ≥ 80yo) Healthy controls (*n* = 13)	≥80yo, normal MMSE for education level, preserved function with CDR 0, no known cognitive impairment, neurologic or psychiatric illness, substance abuse, significant visual or hearing impairment, structural brain lesion on imaging	Structural MRI, MR spectroscopy
Dekhtyar et al 2017^45^	Volunteers enroled in the Harvard ageing brain study, US, Longitudinal 3 years follow up Demographic and imaging factors	‘Optimal memory performers’ (*n* = 25, 77.5 ± 6.75) ‘Typical’ (*n* = 100, 78.89 ± 5.5)	>75yo with no history of serious medical or psychiatric illness, head trauma, substance abuse, normal MMSE and WMS logical memory scores for age and education, CDR 0	Volumetric MRI PET APOE genotype
Dominguez et al 2021^28^	Sample selected from the North American multisite National Alzheimer's Coordinating Centre (NACC) database and the 90 + study cohort (90+) Cross‐sectional single imaging assessment from longitudinal study assessing cingulate cortex and whole brain cortical thickness	Top cognitive performers’ [*n* = 118 74.07 ± 2.68 (NACC) 94.06 ± 2.60 (90+)] ‘Non‐top cognitive performers’ (*n* = 234) 74.34 ± 2.75 (NACC) 93.75 ± 2.62 (90+)	≥70yo with normal cognition and behaviour based on clinician impression, CDR and clinician Judgement of Symptoms (NACC); cognitively normal based on clinician assessment and testing and independent function (90+)	Volumetric MRI
Engelmayer et al., 2023^46^	As per Harrison et al 2012 Cross‐sectional single assessment. Differences in medication profiles in SuperAgers and controls	‘SuperAgers’ (*n* = 96, 82.3 ± 3.4) Cognitively normal controls (*n* = 46, 84.2 ± 4.7)	No neurological or psychiatric illness, preserved function	Nil
Gardener et al 2021^10^	AIBL Longitudinal with assessments at 4 time points over 54 months Serial cortical thickness and volume	‘Superior cognitive performance’ (*n* = 76, 75.58 ± 3.9) Typical older adults (*n* = 100, 76.70 ± 4.4)	As per Dang et al 2019, stable cognition	Volumetric MRI Amyloid PET
Garo‐Pascual et al., 2023^12^	Participants in the Vallecas Project (Spain) longitudinal cohort with up to six yearly follow up. Aim to characterize brain structure and demographic, lifestyle and clinical factors associated with super‐ageing	‘Superagers’ (*n* = 64, median 81.6 IQR 80.4–83.1) ‘Typical older adults (*n* = 55, median 82.1 IQR 81.3–83)	≥79.5, cognitively healthy, no neurological or psychiatric disorders, functionally independent and survival expectancy at least 4 years	APOE genotyping, blood amyloid, tau, glial fibrillary acid protein, neurofilament light, volumetric MRI
Gefen et al 2014^47^	As per Harrison et al 2012 Longitudinal cognitive performance with follow up assessments at 18 months	‘SuperAgers’ (*n* = 18, 82.2 ± 2.4)	No evidence of neurologic or psychiatric disease	Nil
Gefen et al 2015^48^	Northwestern University SuperAging Project and ADNI Cross‐sectional single assessment. Imaging and neuropathological features of the anterior cingulate cortex.	‘SuperAgers’ (*n* = 31, 82.52 ± 2.93) Middle‐aged controls (*n* = 18, 58.39 ± 3.7), older adult controls (*n* = 21, 83.76 ± 4.0)	Nil clinical or imaging evidence of neurologic or psychiatric disease, intact daily functioning	Volumetric MRI APOE genotype Neuropathology
Gefen et al., 2021^24^	As per Harrison et al 2012 Neuropathological examination entorhinal cortex SA	‘SuperAgers’ (*n* = 7, 89.9 ± 5.8) Controls (*n* = 6, 87.8 ± 7.4)	Controls required to be cognitively normal prior to death based on chart review	Neuropathology
Harrison et al 2012^5^ ^,^ ^a^	Community‐dwelling volunteers recruited through clinics, lectures and word of mouth to the US Northwestern University SuperAging Project Cross‐sectional single assessment, cortical volumes	‘SuperAgers’ (*n* = 12, 83.5 ± 3.0) Middle aged controls (*n* = 14, 57.9 ± 4.3) and older adult controls (*n* = 10, 83.1 ± 3.4)	≥70yo with no neurologic, psychiatric or major medical illness, no medications affecting cognition, MMSE ≥25	Volumetric MRI
Harrison et al 2018^49^	Volunteers from the US Berkeley ageing cohort study Longitudinal with average 5 years cognitive follow up data and follow up MRI in a subset of participants. Cortical thickness, hippocampal volume, WMH and amyloid.	‘Successful agers’ (*n* = 26, 74.9 ± 4.6) Typical older adults (*n* = 103, 75.9 ± 4.5)	≥80yo, no cognitive impairment	Volumetric MRI Amyloid PET APOE genotype
Hoenig et al., 2020^50^	ADNI database. Cross‐sectional single assessment. Differences in PET amyloid and tau	‘Super agers’ (*n* = 25, 85.2 ± 3.5) ‘Normal agers’ (*n* = 25, 84.5 ± 3.5) MCI (*n* = 25, 84.8 ± 3.9) Younger controls (*n* = 19, 63.6 ± 2.8)	≥80yo	Amyloid and tau PET
Huentelman et al 2018^18^	Northwestern University SuperAging Project and ADNI Cross‐sectional single assessment, genetic variation	‘SuperAgers’ (*n* = 56, 83.0 ± 3.3) Cognitively average controls (*n* = 22, 82.8 ± 2.6)	No evidence of neurologic or psychiatric disease	Genotyping
Janeczek et al 2018^51^	Brain tissue obtained from neuropathologists across the US and the Northwestern University Alzheimer's disease Centre brain Bank Cross‐sectional single assessment	‘SuperAgers’ (*n* = 5, 90.2) NC (*n* = 27, range of ages)	Right‐handed, native English speaking, no significant visual impairment, substance abuse or neurologic or psychiatric disorder; normal performance on cognitive screening tests	Neuropathology with acetylcholinesterase staining
Karpouzian‐Rogers et al., 2022^52^	As per Harrison et al 2012 Determining if SA also had superior non‐verbal memory Cross‐sectional single assessment	‘SuperAgers’ (*n* = 46, 84.2 ± 3.3) Cognitive average for age (*n* = 31, 84 ± 3.9)	No significant neurological or psychiatric illness, preserved functioning	Nil
Katsumi et al 2021^53^	North American volunteers from the Boston area participating in the Massachusetts general Hospital brain resilience in ageing: Integrated Neuroscience (BRAINS) programme Cross‐sectional single assessment, task‐based fMRI	‘Superagers’ (*n* = 17, 67.8 ± 6.0) Young controls (*n* = 41, 24.5 ± 3.6), typical older adults (*n* = 40, 66.2 ± 5.1)	≥60yo, normal cognition, no neurologic or psychiatric illness, significant visual or hearing impairment, major medical issue or medication that could affect cognitive function	Structural and functional MRI
Katsumi et al., 2022^16^	Elective surgery patients from Harvard University (US) affiliated medical centres enroled in the successful ageing after Elective Surgery study, followed up at least 6 months Single pre‐operative neuropsychological assessment Post‐operative delirium in SA and association with cortical thickness	‘SuperAgers’ (*n* = 19, 75.5 ± 4.5) Delirious typical older adults (*n* = 15, 75.7 ± 4.3) Non‐delirious typical older adults (*n* = 59, 76 ± 4)	≥70yo undergoing elective surgery with an anticipated length of stay ≥3 days, no delirium dementia or hospitalization within 3 months prior to participation, terminal condition, blindness, severe deafness, history of psychotic illness or alcohol abuse	Volumetric MRI
Kim et al 2020^20^	Community dwelling volunteers with normal cognition recruited from a dementia prevention centre in Seoul, Korea Cross‐sectional single assessment, imaging white matter integrity	‘SuperAgers’ (*n* = 35, 71.0 ± 5.3) Typical agers (*n* = 55, 73.0 ± 5.6)	Healthy controls required to be cognitively normal, MCI on basis of psychiatrist or neurologist diagnosis	MRI with diffusion tensor imaging
Lin et al 2017^54^	Sample derived from ADNI dataset Longitudinal with at least one follow‐up assessment Relationships between amyloid deposition and brain connectivity	‘Supernormals’ (*n* = 9, 73.53 ± 6.38) Healthy controls (*n* = 9, 72.31 ± 5.57), MCI (*n* = 9, 72.97 ± 6.91)	68‐74yo at study commencement with no history of stroke and MMSE ≥24	Functional MRI Florbetapir PET
Maccora et al 2020^13^	Random recruitment from electoral rolls of Australian adults in the Australian Capital Territory and Queanbeyan for the Personality and Total health through life (PATH) cohort study Longitudinal with 3 waves of follow up approximately 4 years apart Prevalence, clinical and demographic factors associated with SA	‘SuperAgers’ (*n* = 116, 70.42 ± 1.58 F 70.24 ± 1.35 M) NC (*n* = 1563) 70.68 ± 1.52 F 70.55 ± 1.48 M	≥70yo, English speaking, no significant vision or hearing impairment, neurologic or psychiatric illness, use of medications likely to affect cognition, serious blood disorder	APOE genotype
Mapstone et al 2017^55^	Volunteers recruited to the North American Rochester/Orange County ageing study (R/OCAS) Longitudinal with yearly blood tests and cognitive assessments	‘Supernormal’ (*n* = 41, 83.22 ± 3.37) NC (*n* = 41, 83.29 ± 3.82)	Cognitively normal, no neurologic or psychiatric disease and preserved function	Blood metabolomics
Nassif et al., 2022^56^	As per Harrison et al 2012 Neuropathology: Entorhinal cortex integrity in SA	‘SuperAgers’ (*n* = 6, 91.3 ± 5.7) Normal elderly (*n* = 7, 89.3 ± 4.8) Amnestic MCI (*n* = 5, 92.4 ± 3.9) Younger controls (*n* = 6 ± 13)	As per Harrison et al 2012	Neuropathology
Park et al., 2022^21^	Community dwelling volunteers recruited from two dementia prevention centres in Seoul, Korea. fMRI functional connectome of SA and machine learning‐based predictive models. Cross‐sectional	‘Superagers’ (*n* = 58, 72.8 ± 5.5) Typical agers (*n* = 32, 71 ± 5.5)	≥60 yo literate, no cognitive impairment, neurological or psychiatric condition, significant visual or hearing impairment, medications affecting cognition or other major medical condition	Volumetric and functional MRI
Rogalski et al 2013^17^	As per Harrison et al 2012 Cross‐sectional single assessment. Structural neuroimaging, genetic and pathologic features of SA	‘SuperAgers’ (*n* = 12, 83.5 ± 3.0) Middle‐aged controls (*n* = 14, 57.9 ± 4.3), older adult controls (*n* = 10, 83.1 ± 3.4)	As per Harrison et al 2012	Structural MRI APOE genotype Neuropathology
Rogalski et al 2019^23^	As per Harrison et al 2012 Longitudinal, at least 3 years follow up. Autopsy study.	‘SuperAgers’ (*n* = 10, 85.5)	As per Harrison et al 2012	APOE genotype Neuropathology
Saint Martin et al 2017^14^	Sample derived from the French Evaluation of ageing, Autonomic nervous system activity and Cardiovascular events (PROOF) study; originally random selection from electoral rolls Longitudinal with baseline and follow‐up assessments 8 years later. Cognitive trajectories and lifestyle in later life.	‘Cognitively elite’ (*n* = 186, 66.9 ± 0.9^b^) Cognitively normal (*n* = 331, 74.8 ± 1.0), cognitively impaired (*n* = 23, 74.8 ± 0.9)	No neurologic condition, language barrier or significant visual impairment	Nil
Spencer et al., 2022^57^	Northwestern SuperAging Programme and control group from the University of California, San Diego Shiley‐Marcos Alzheimer's disease research Centre Alzheimer's genetic risk scores in SA. Cross‐sectional.	‘SuperAgers’ (*n* = 37, 82.7 ± 2.8) Controls (*n* = 35, 83.7 ± 4.3)	SA as per Harrison et al 2012 Controls ≥80yo with average performance for age on cognitive measures	Blood genetic testing including APOE
Sun et al 2016^19^	Paid participants recruited from the Boston area, North America Cross‐sectional single assessment Cortical thickness in the default and salience networks	‘Superaging’ (*n* = 17, 67.8 ± 6.0) Young adults (*n* = 41, 24.5 ± 3.6), typical older adults (*n* = 23, 66.2 ± 5.1)	No neurologic or psychiatric disorder, substance dependence, CNS‐active medications; native English speaking with normal/corrected normal vision	Structural and functional MRI
Wagner et al., 2022^58^	Female registered nurses enroled in the Nurses' health study (US) 30 years longitudinal study assessing impact of lifestyle factors on cognition	‘Exceptional episodic memory’ at ≥80yo (*n* = 455, 61.5^c^ ± 1.7) Cognitively average matched controls (*n* = 2275, 61.8 ± 1.7)	≥70yo with no history of stroke	Nil
Yang et al., 2022^11^	Community dwelling participants enroled in the Beijing Ageing brain Rejuvenation Initiative cohort study Cross‐sectional single assessment Early and late life lifestyle and clinical factors associated with cognition	‘Successful cognitive ageing’ (*n* = 154, 73.8 ± 3.8) Cognitively normal controls (*n* = 173, 74.8 ± 3.9) MCI (*n* = 256, 76.1 ± 4.2)	≥70yo, ≥24 on the Chinese MMSE, no severe neurological or psychiatric condition, no psychoactive medications	Nil
Yu et al 2020^59^	Volunteers recruited through door‐to‐door visits by nurses within districts of western Singapore Cross‐sectional single assessment Lifestyle factors associated with SA	‘Super‐cognition’ (n = 64, 67.3 ± 5.6) NC (n = 263, 67.4 ± 5.2)	No neurologic or psychiatric diagnosis, cognitive impairment, CDR = 0	Nil
Zhang et al 2020^60^	Volunteers recruited from the Boston area, North America Cross‐sectional single assessment fMRI connectivity within the default mode and salience networks	‘Superagers’ (*n* = 17, 67.8 ± 6.0) Young adults (*n* = 41, 24.5 ± 3.6), typical older adults (*n* = 40, 66.9 ± 5.5)	No neurologic or psychiatric disorder, normal or corrected normal vision, native English speakers	Structural and functional MRI

Abbreviations: 90+: The 90 + Study cohort, AD: Alzheimer's disease, ADNI: Alzheimer's Disease Neuroimaging Initiative, APOE: apolipoprotein E, CNS: central nervous system, CPS: cognitive processing speed, CR1: complement receptor 1, FDG: fluorodeoxyglucose, fMRI: functional MRI, F: female, M: male, MCI: mild cognitive impairment, MMSE: Mini‐Mental State Examination, MoCA: Montreal Cognitive Assessment, MRI: magnetic resonance imaging, NACC: National Alzheimer's Coordinating Centre, NC: normal controls, PET: positron emission tomography, SD: standard deviation, SE: standard error, SA: SuperAgers, WMH: white matter hyperintensities.

^a^ Hereafter referred to as Harrison et al 2012 definition.

^b^ Mean age of all participants, not just cognitively elite.

^c^ Age at baseline assessment in 1986.

**TABLE 2:** Components of published super‐ageing definitions.

Term (study)	Population	Cognitive domain/s	Tests	Cut‐offs
SuperAgers				
Borelli et al 2021, Cervenkova et al 2020, Cook et al 2017, Cook Maher et al 2017, Cook Maher et al 2022, Engelmayer et al 2023, Harrison et al 2012, Gefen et al 2014, Gefen et al 2015, Gefen et al 2021, Huentelman et al 2018, Janeczek et al 2018, Karpouzian Rogers et al 2022, Nassif et al 2022, Rogalski et al 2013, Rogalski et al 2019, Spencer et al 2022	≥80	Verbal episodic memory Executive function, verbal fluency, language/naming	RAVLT delayed recall TMT‐B, CFT, BNT‐30	≥9/normative mean for 50‐65yo At least average (≥‐1 SD for age and education)
High performance older adults Carmona et al 2023	Mean age 75	Verbal episodic memory Global cognition, visual memory, category fluency	RAVLT delayed recall MMSE, BCSB visual memory and category fluency	≥9/normative mean for 50‐65yo ≥ Age norms and no impairment on neuropsychological assessment^a^
Successful healthy agers Bezdicek et al 2020	Mean age 75	Verbal episodic memory	PVLT delayed recall	≥9/normative mean for 50‐65yo
		Executive function, verbal fluency, language/naming	TMT‐B, CFT, BNT‐30	≥‐1 SD for age and education
		Global cognition	MoCA	Non‐negative random slope over 5 years
Superagers				
de Godoy et al 2021	≥80yo	Verbal episodic memory	RAVLT delayed recall	≥9/normative mean for 50‐65yo
		Executive function, attention, working memory, visuospatial, verbal fluency, language/naming	TMT‐A and B, digit span, RCFT, category and letter fluency, BNT‐60	≥ −1 SD for age and education
Harrison et al 2018, Katsumi et al 2021, Sun et al 2016, Zhang et al 2020	Mean age 68	Verbal episodic memory	CVLT long delay free recall	≥ gender‐adjusted normative mean for 18‐32yo
		Executive function	TMT‐B	≥ −1 SD mean for age
Katsumi et al 2022	Mean age 76	Verbal episodic memory	HVLT	≥ gender‐adjusted normative mean for 16‐29yo
		Executive function	TMT‐B	≥ −1 SD mean for age
SuperAgers				
Maccora et al 2020	Mean age 70	Verbal episodic memory	CVLT immediate and delayed recall	Maintaining scores ≥ median for those of the same gender in their 20s
		Global cognition	MMSE	≥29 over 3 waves of follow up
Dang et al 2019	Mean age 68	Verbal episodic memory	CVLT delayed recall	≥ Normative mean for 30‐40yo
		Executive function, working memory, verbal fluency	Digit symbol substitution, Stroop, digit span, letter and category fluency	≥ −1 SD mean for age
Kim et al 2020, Park et al 2022	Mean age 71	Verbal episodic memory	SVLT	≥ Average normative values for 45yo
		Visual memory	RCFT	
Superagers				
Garo‐Pascual et al 2023	Mean age 82	Verbal episodic memory Executive function, verbal fluency, language/naming	Free and Cued Selective Reminding test Digit Symbol Substitution test, CFT, BNT‐15	≥ Normative mean for 50‐56yo ≥‐1 SD for age + Maintain performance over up to 5 years follow‐up
High‐performing older adults				
Cabeza et al 2002	Mean age 68	Memory composite	Logical memory, verbal paired associates, CVLT long‐delay cued recall	Average standardised score comparable to young adults 20‐35yo (standardised mean 0.62 vs. 0.54)
Superior cognitive performer				
Gardener et al 2021	Mean age 76	Verbal episodic memory Executive function, working memory, verbal and visual memory, verbal fluency, language/naming	CVLT delayed recall Digit span, digit symbol coding, Stroop, logical memory, RCFT delay, letter and category fluency, BNT	Z scores within 0.5 of 30‐44yo Z scores within 1.5 for age For ≥3 time points up to 54 months
Resilient‐agers				
Bott et al 2017	Mean age 69	Cognitive processing speed	Computerised test	Scores within 1.25 SD of young adult comparator group in their 20s and <0.5 SD change at follow up (mean 2.5 years)
Supernormals				
Lin et al 2017	Mean age 74	Episodic memory composite	MMSE, ADAS‐Cog, RAVLT, logical memory	≥1.5 SD of sample across available study visits (≥1 follow up)
Super agers				
Hoenig et al 2020	Mean age 85	Episodic memory composite	ADNI‐MEM (RAVLT, ADAS‐Cog, MMSE, logical memory test)	Mean Z scores ≥1.25 over 4 years
Supernormal				
Mapstone et al 2017	Mean age 83	Verbal episodic memory Global cognition, executive function, attention, verbal fluency, language/naming, visuospatial	RAVLT learning, retrieval and recognition composite MMSE, TMT‐A and B, CFT, BNT‐60, forward and backward digit span, HVOT	Scores ≥90th percentile of sample > −1.35 or >10th percentile all other domain composite scores
Supernormals				
Baran and Lin 2018	Mean age 74	Episodic memory composite Executive function composite Function	ADNI‐MEM ADNI‐EF (digit symbol Substitution, digit Span backwards, TMT‐A And B, CFT, CDT) CDR	Mean Z scores 1.51 for memory 1.05 for executive function compared with normal controls and scores ≥1 SD above normal population mean with consistently high performance over 5‐year period 0
Optimal performers				
Dekhtyar et al 2017	Mean age 78	Composite memory	Memory capacity test, face name associative memory exam, selective reminding test	Performance in the top 20% with maintenance at 3‐year follow up
Exceptional memory				
Wagner 2022	≥80yo women	Verbal episodic memory	TICS 10‐word list immediate and delayed recall, EBMT immediate and delayed recall	Composite score ≥1.5 SD sample mean
Top cognitive performance				
Dominguez et al 2021	2 cohorts: Mean age 74, 94	Verbal episodic memory and executive function	Logical memory delayed and TMT‐B for ≤90yo, CVLT delayed recall and TMT‐B for ≥90yo	Within the top 50th percentile of the sample for younger group, within the top 50% expected for age for older group
Successful cognitive ageing				
Yang et al 2022	Mean age 74	Verbal episodic memory	RAVLT delayed recall	>1.5 SD age and education adjusted sample mean for either
		Executive function	TMT‐B	
		General cognition, memory, visuospatial ability, attention, executive function	MMSE, RAVLT, complex figure test copy and delay, CDT, symbol digit test, TMT‐A and B, Stroop Colour and word test	≥ −1.5 SD for all
Cognitively elite				
Saint‐Martin et al 2017	Mean age 67	Composite information processing and attention Composite executive function Composite memory	TMT‐A, Stroop part 1 and 2, WAIS coding subtest TMT‐B, Stroop part 3, letter and category fluency, WAIS similarities MMSE, BVRT, Grober and Buschke Selective reminding test	Performance above the sample mean for all Follow up: stable, worsened or improved
Super‐cognition				
Yu et al 2020	Mean age 67	RBANS: Immediate memory, visuospatial, language, attention, delayed Function	Word list learning, story, figure copy, line orientation, picture naming, category fluency, digit symbol test, list recall, list recognition, delayed recall story, figure recall CDR	Score ≥1 SD above age and education appropriate norms in ≥1 domain and ≥average performance in all other domains 0
Superior global cognitive performers				
Biswas et al 2023	Mean age 97	Global cognition (and normal diagnosis in study consensus conference)	MMSE	≥28 at last visit 2–12 months before death

Abbreviations: ADAS‐Cog, Alzheimer’s Disease Assessment Scale – Cognitive Subscale; ADNI EF, Alzheimer’s Disease Neuroimaging Initiative composite executive function score; ADNI‐MEM, Alzheimer’s Disease Neuroimaging Initiative composite memory score; BNT‐30, 30‐item Boston Naming Test; BNT‐15, 15‐item Boston Naming Test; BNT‐60, 60‐item Boston Naming Test; BVRT, Benton Visual Retention Test; CDR, Clinical Dementia Rating scale; CDT, Clock Drawing Test; CFT, Category Fluency Test; CVLT, California Verbal Learning Test; EBMT, East Boston Memory Test; fMRI, functional magnetic resonance imaging; H‐MR, proton magnetic resonance spectroscopy; HVLT, Hopkins Verbal Learning Test; HVOT, Hooper Visual Organisation Test; MMSE, Mini‐Mental State Examination; MoCA, Montreal Cognitive Assessment; MRI, magnetic resonance imaging; NIHTB, National Institutes of Health Toolbox; PiB‐PET, Pittsburgh compound B positron emission tomography; PVLT, Philadelphia Verbal Learning Test; RAVLT, Rey Auditory Verbal Learning Test; RBANS, Repeatable Battery for the Assessment of Neuropsychological Status; RCFT, Rey Complex Figure Test; SD, standard deviation; SVLT, Seoul Verbal Learning Test; TICS, Telephone Interview for Cognitive Status; TMT‐A and B, Trail Making Test Parts A and B; WAIS‐R, Wechsler Adult Intelligence Scale‐Revised; WMS‐R, Wechsler Memory Scale‐Revised; WTAR, Wechsler Test of Adult Reading.

^a^ Only a random subset of healthy individuals underwent detailed neuropsychological testing in this study.

### 3.1 Summary of included studies

The clinical populations of included studies mostly came from large longitudinal cohorts based in North America (*n* = 30) with a smaller number from Europe (*n* = 4), Brazil (*n* = 3), Australia (*n* = 3), Korea (*n* = 2), China (*n* = 1) and Singapore (*n* = 1). Twenty‐two studies (50%) used convenience samples drawn from existing longitudinal ageing studies. The remaining studies prospectively recruited participants including the longitudinal Northwestern University SuperAging Project from which 14 studies (32%) drew data. While all studies concerned older adults, the ages of participants were diverse. Twenty‐three samples (52%) involved those aged 80 and over, thirteen (30%) included those with a mean age in their 70s and eight (18%) in their late 60s.

Inclusion criteria were relatively uniform across studies. The majority required the absence of an established neurologic or psychiatric disorder. There was variability in how detailed other exclusions were with stroke, cognitive impairment, substance abuse, other serious medical illnesses, medications and other treatments that could impair cognition including chemo and radiotherapy, visual and hearing impairments, literacy and first language other than testing language variously reported.

Most studies (*n* = 35, 80%: 14 from the Northwestern University SuperAging Project, 21 others) defined super‐ageing in relation to verbal episodic memory performance. Only three studies (7%) explicitly included a test of visual memory in definitions. Bott et al were the first to characterize a group of older adults who maintain cognitive processing speed with age.^27^ Two other studies used performance in a single cognitive domain other than episodic memory to define super‐ageing. Yu et al required older adults with ‘super‐cognition’ to have scores at least 1 standard deviation above age and education‐appropriate norms in at least one cognitive domain assessed in the Repeatable Battery for the Assessment of Neuropsychological Status (RBANS).^59^ Yang et al determined that a score higher than 1.5 standard deviations above the mean on either a verbal episodic memory task or executive function task constituted ‘successful cognitive ageing’.^11^ Others have used composite or global cognitive measures. Baran et al used composite episodic memory and executive function scores approximately 1 standard deviation above the normal population mean.^34^ Saint Martin et al defined a ‘cognitively elite’ group on the basis of Z scores above the mean for the three composite cognitive domains of information processing speed and attentional performance, executive function and memory.^14^ Few studies included a measure of global cognition such as the MMSE in their super‐ageing definition (*n* = 4, 9%) although most (*n* = 31, 71%) required that high‐performing individuals demonstrate at least average performance in cognitive domains aside from memory. Biswas et al were the only group who relied solely on a global cognition measure (MMSE) to define super‐ageing in a cohort over the age of 90.^36^

Most studies (*n* = 33, 75%) defined exceptional performance in relation to younger adult norms with comparator groups ranging in age from 16 to 65. Many (*n* = 21, 14 from the same group) studies used the Harrison et al cut‐off of episodic memory performance expected for 50–65‐year‐olds.^5^ Four studies compared performance to 18–32‐year‐olds, one study to 16–29‐year‐olds, two to those in their 20s and four to those in their 30 and 40s. Bott et al compared the cognitive processing speed performance of over 60‐year‐olds with young adult controls, mean age 24. Eight studies determined super‐ageing based on episodic memory and/or other cognitive domain scores 0.5–1.5 standard deviations above the population mean. One study selected performers in the top 20th percentile on a memory composite score^45^ and one study those in the 50th percentile for episodic memory and executive function.^28^

Most studies relied on cross‐sectional assessment of cognition with only nine requiring maintenance of exceptional performance over time and one study further characterising participants by stable, worsened or improved performance at follow up.^14^ Baran et al required ‘supernormals’ to maintain a positive mean slope of executive function and episodic memory performance over a 5‐year period.^34^ Bezdicek et al required a non‐negative random slope of Montreal Cognitive Assessment scores over 5 years in ‘successful healthy agers’.^35^ Bott et al determined that ‘resilient‐agers’ have 0.5 SD or less change in cognitive processing speed at follow up (median 2.5 years).^27^ Dekhtyar et al compared those who maintained performance on a memory composite score at 3 years versus those who declined.^45^ Gardener et al applied their criteria to all time‐points assessed up to 54 months^10^ Garo‐Pascual required ‘superagers’ to maintain their classification at up to 5 years follow‐up.^12^ Hoenig et al required maintenance over 4 years,^50^ Lin et al required ‘supernormals’ to have episodic memory composite scores above 1.5 across all available assessments with at least one follow‐up assessment^54^ and Maccora defined their group as those who maintained episodic memory performance above that of adults in their 20s and a MMSE score 29 or above over 3 waves of follow up.^13^

In terms of other measures of successful ageing, only Baran et al and Yu et al specifically incorporated a CDR of 0 and independent daily functioning respectively into their super‐ageing definitions.^34^, ^59^ However, 12 other studies required that all participants including comparator groups have evidence of intact function with or without the use of a specific rating scale such as the CDR to establish this. Six studies reviewed associations with lifestyle factors, four studies specifically reviewed physical activity levels and another assessed psychological wellbeing. The majority of studies (*n* = 32) examined laboratory and/or imaging biomarkers associated with super‐ageing; neuropathologic autopsy data were available in nine.

Three additional studies were identified that classified super‐ageing on the basis of post‐hoc statistical modelling of baseline and follow‐up cognitive performance in a cohort of older individuals to identify a cluster of people with superior cognition. Chen et al used latent mixture modelling to identify different classes of cognitive performance with ‘successful agers’ demonstrating superior cognitive performance and better preserved performance over time across four domains (episodic memory, inductive reasoning, working memory, speed of processing).^61^ Klinedinst et al used cluster analysis to identify ‘super‐agers’ aged 45–76 with either slightly positive or stable fluid intelligence trajectories over 10 years^62^ and Lin et al had previously used a latent class analysis to identify a subgroup of ‘successful cognitive agers’ who exhibited high stable episodic memory and executive function over 5 years of follow‐up.^63^ While this approach was noteworthy, these studies were not included in this review as they did not provide a specific a priori definition of super‐ageing.

### 3.2 Methodological quality of the included studies

Two studies provided very little information on how cases and controls were selected and if they were matched appropriately. However, one of these studies was published as a letter with presumably a more stringent word count and limitations on tables and was part of a broader super‐ageing study which included these details in other publications. Demographic and clinical characteristics of cases and controls differed markedly in nine studies. More females were included in cognitively high‐performing than control cohorts in six of these studies^11^, ^18^, ^19^, ^28^, ^49^, ^53^ while education levels were significantly higher in six cognitively high performing cohorts compared with controls.^11^, ^12^, ^13^, ^15^, ^28^, ^49^ Rates of hypertension and diabetes were higher in the cognitively average than the super‐ageing cohort in one study but not significantly so.^43^ These differences may represent selection bias in some studies. Statistical analysis was performed to detect group differences in all cases.

## 4 DISCUSSION

Existing definitions of cognitive super‐ageing vary widely in terms of the demographics of samples and comparator groups as well as the cognitive criteria used. However, they have generally included cross‐sectional assessments of relatively older individuals with a focus on episodic memory performance. The components of published definitions are summarized in Table 2. There has been little research thus far concerning the interactions between exceptional cognition in older age and other aspects of ageing well such as physical super‐ageing.

Definitions of super‐ageing have included different cognitive measures which, to varying degrees, reflect changes in the ageing brain. Ageing is associated with a general reduction in cognitive capacity but there is considerable variability in trajectories between individuals and how much decline is attributable to neuropathology remains an active area of research. Super‐agers illustrate human potential to maintain high level cognitive performance at advanced ages and the degree that cognitive decline may be potentially preventable. Typically, cognitive processing speed, aspects of memory including working memory and episodic memory, verbal fluency and conceptual reasoning which are aspects of so‐called fluid intelligence tend to decline with age.^64^, ^65^, ^66^ Processing speed has been shown to decline from the third decade of life while memory performance may begin to decline in the 50s and executive function especially after age 70 but more complex reasoning capabilities beginning around age 45.^65^, ^67^ Conversely, crystallised abilities including vocabulary, general knowledge and spatial perception tend to remain intact or improve through the sixth and seventh decades of life.^66^, ^67^ Similarly, wisdom which refers to the amount of knowledge an individual has accumulated over their lifespan and how they are able to use and apply it in the mastery of life matters, may increase then remain stable in older adulthood.^64^ Older adults may have either comparable or greater capacity for emotional regulation than younger adults.^68^

Interestingly, analyses of the cognitive profiles of older adults with exceptional verbal episodic memory revealed that they also demonstrated better performance than average memory controls on measures of non‐verbal episodic memory, working memory and attention but not across other domains including processing speed, executive function, language and visuospatial measures.^42^, ^52^ Verbal episodic memory is vulnerable to ageing but concentrating on exceptional performance in this or other single cognitive domains especially at a single time point may provide limited insight into processes of cognitive ageing in super‐agers.

Similarly, the cognitive domains included in existing super‐ageing definitions do not reflect the spectrum of neuropathology including AD, other neurodegenerative pathologies and cerebrovascular disease that accumulates with age. One or more pathologies are evident in most individuals over age 80^69^ but differentially impact upon cognition. Preclinical and prodromal stages of AD typically extend over 20 years^69^ with subtle declines in cognitive performance including memory before the development of overt dementia. Similarly mild impairments in executive and visuospatial function are seen in the prodrome of dementia with Lewy bodies.^70^ Large neuropathological studies have related Alzheimer's pathology to measures of global cognition, episodic memory, working memory, perceptual speed and visuospatial ability; Lewy body pathology most closely to visuospatial impairments and cerebrovascular pathology to global cognition in the case of high volume large infarcts and small vessel disease having a weaker association with fluency, episodic memory, semantic memory and perceptual speed.^71^

The clinical hallmark of AD is episodic memory impairment^72^, ^73^ and thus studying individuals with preserved episodic memory may eliminate those with early typical AD while potentially missing the impact of other evolving neuropathologies such as frontotemporal lobar degeneration with its effects on executive function and language abilities and Parkinsonian disorders with associated visuospatial impairment.^72^, ^73^ Using composite scores comprising one or a couple of cognitive domains to define super‐agers may be similarly limited.

The association between burden of neuropathology and cognition is not consistent. A study of over 1000 older people who had annual cognitive testing for up to 24 years and underwent post‐mortem neuropathologic examination demonstrated no residual cognitive decline in most individuals when neuropathologic effects were accounted for.^74^ Borland et al showed that when older individuals with evidence of pathology on the basis of cerebrovascular changes on MRI or cerebrospinal fluid (CSF) amyloid, P‐tau and neurofilament light were excluded, age‐related effects on cognitive test results disappeared.^75^ However, other studies have not been able to attribute variability in rates of cognitive decline solely to neuropathology.^76^, ^77^ Boyle et al demonstrated that in over 1000 people followed with annual cognitive assessments and neuropathological assessment at death, there was considerable heterogeneity in cognitive trajectories and while almost all had evidence of neuropathology, pathological indices accounted for only 43% of the variance in cognitive decline.^76^ Super‐agers are an ideal group to study to determine the presence or absence of other as yet unmeasured pathological processes, resilience factors and mechanisms of compensation which may underly this unexplained variability. An approach that combined assessment of global cognition as well as performance in all individual cognitive domains may provide greater insights into age trajectories of different cognitive processes and the impacts of neuropathology.

Considering the heterogeneity in cognitive capacity of older adults and their cognitive trajectories, identifying those with superior performance at any one point in time may not predict maintenance of cognition. Factors such as genetics and experiences over the lifespan including level of education and adverse health and social events affect the baseline cognitive abilities of older adults.^64^ A lack of cognitive decline over time would be more meaningful to most individuals than comparison of performance to other older adults.^78^ So far, maintenance of cognitive capacity has been captured in several definitions of super‐ageing but only in regard to those who already displayed superior performance in one or a couple of cognitive domains. Longitudinal follow up of such individuals will likely continue to provide important insights.

The demographics of super‐ageing samples and their comparator groups have varied widely. Those aged 80 and above are more likely to manifest cognitive impairment than those in their 60s or 70s which is why many studies have restricted their samples to this group.^5^ Individuals aged 75 and above have been selected in low‐ and middle‐income countries.^15^, ^26^ However, this approach may limit research into the longitudinal trajectories of cognition in super‐agers. Defining exceptional cognition in older adults relative to their age peers is a logical distinction and useful when a comparison is being made between good and poor cognitive functioning. However, given cognitive performance typically declines with age, such individuals may be performing better than others their age but still have a degree of cognitive impairment. Comparing cognitive performance with average performance of adults in mid‐life, 50–65 years old, has practical relevance given the majority of such individuals are likely still working and functioning independently in society. However, brain health at this age is dependent on a range of socioeconomic circumstances and the presence of comorbidities.^79^, ^80^ Additionally, adults within this age range may have prodromal neurodegenerative disease. Comparisons with healthy younger adults effectively ensures that an assessment is being made against peak performance. However, elements of cognition change with age and it is perhaps unrealistic that an older adult would demonstrate equivalent processing speed to a 20‐year‐old or expect comparative cognitive performance more broadly.

Klinedinst et al^62^ and others have employed statistical modelling to identify subgroups of older individuals within longitudinal ageing studies who demonstrate both superior cognitive performance and maintenance of cognitive abilities. This data‐driven approach represents an attractive solution to the arbitrary nature of using pre‐defined cut‐offs for cognitive performance and provides further insights into the cognitive profiles of those maintaining high cognitive performance. Future similar studies would be very valuable for this field. While they do not at this stage provide a reproducible method of prospectively identifying super‐agers, insights from such studies may better inform future selection of cognitive measures of super‐ageing. For example, super‐agers in these studies displayed above average executive function with maintenance of performance over time. Most prior studies have only required average for age performance on an executive function task with a minority incorporating superior executive function into their definitions.^11^, ^14^, ^28^, ^34^

This review has illustrated a range of approaches to defining super‐ageing. Ultimately, multiple definitions may be fit for purpose. For example, it may be relevant to identify older adults with comparatively better cognitive function than their age peers in some contexts, acknowledging that a degree of cognitive decline is inevitable with age whereas stricter definitions involving performance comparable to younger adults allows for a smaller more remarkable group to be identified. These groups may overlap as people who perform as well as younger adults are also likely to outperform their peers. Selecting individuals with exceptional performance in individual cognitive domains may provide further insights into cognitive ageing but the narrow focus of these definitions should be recognised. Similarly, the limitations of cross‐sectional identification of top cognitive performers must be acknowledged. Categorising super‐ageing purely on the basis of a lack of cognitive decline over time is an attractive proposition to deal with baseline heterogeneity in the cognitive abilities of older adults. Regardless of the approach employed, clearly operationalized criteria should be utilized along with a rationale for the choice of criteria, relevant to the research question.

Future research may involve the development of a differential model examining performance within individual cognitive domains as well as global cognitive performance over time and may provide further insights into those functions crucial for adaptation to ageing and neurodegeneration. Defining exceptional cognition in older adults both in relation to their age peers and to the average performance of younger adults may overcome issues associated with comparison to either group alone. The relationship between superior cognition and daily functioning also warrants greater attention. Future studies may incorporate rating scales for independence in activities of daily living and elucidate practical aspects of successful cognitive ageing including compensation for deficits and personal control. Associations with other aspects of ageing well such as preserved physical capacity and social engagement would be similarly valuable. Super‐ageing is more likely to remain a research concept that could be integrated into broader successful ageing measures and identify predictors of sustained high‐level cognitive function.

While not specifically addressed in this review, multiple super‐ageing studies have used laboratory and imaging measures to identify differences in super‐agers including blood metabolomic profiles, whole brain and regional brain volumes, functional imaging and white matter integrity measures. The brain ageing research literature may also be a source of useful biomarkers such as the imaging BrainAGE algorithm.^81^ Future refined definitions of super‐ageing should consider a combination of cognitive measures and these types of biomarkers.

### 4.1 Limitations

A strength of this review was the broad and comprehensive search strategy allowing for the incorporation of diverse definitions of super‐ageing. Limitations included the exclusion of grey literature or unpublished studies which can counterbalance publication bias and studies not published in English which could result in an over‐representation of Western cultural definitions of super‐ageing.

### 4.2 Conclusion

This review demonstrates the heterogeneity of existing cognitive super‐ageing definitions. Differences are extensive including the ages of the super‐ageing cohorts and comparator groups, cognitive domains and tests selected and whether performance is compared with age peers or younger adults. Maintenance of cognitive abilities over time is inconsistently required and there has been limited focus on superior cognitive performance in domains other than verbal episodic memory when other cognitive processes are known to be susceptible to cognitive ageing. A future approach could be to apply different criteria to identify groups of super‐agers from a large population sample. If different definitions identify different populations, it may be instructive to compare how each definition performs against an external criterion such as function or maintenance of cognitive performance. Examination of how cognitive super‐ageing relates to physical capacity, psychological wellbeing and degree of social engagement may also provide greater insights into ageing well. Harmonisation of criteria within the field could lead to opportunities for collaboration, incorporation into research on the broader multidimensional construct of successful ageing and ultimately the opportunity to better understand the aetiology of super‐ageing and ramifications for the general population.

## AUTHOR CONTRIBUTIONS

Alice Powell was involved in conceptualization of the review, methodology, data curation, analysis and writing—original draft. Zara A Page was involved with data curation and analysis. Perminder S Sachdev was involved in conceptualization, methodology, supervision and writing—review and editing. Jacqueline CT Close was involved in conceptualization, methodology, supervision and writing—review and editing. Henry Brodaty was involved in conceptualization, methodology, supervision, writing, review and editing.

## CONFLICT OF INTEREST STATEMENT

PS has been a member of the expert advisory committee for Biogen and Roche. HB has been an advisor or consultant for Biogen, Nutricia, Roche and Skin2Neuron. The other authors have no competing interests to declare.

## Data Availability

The data that support the findings of this study are available from the corresponding author upon reasonable request.
